# A Numerical Assessment of Mitigation Strategies to Reduce Local Oxygen and Proton Transport Resistances in Polymer Electrolyte Fuel Cells

**DOI:** 10.3390/ma16216935

**Published:** 2023-10-28

**Authors:** Pablo A. García-Salaberri

**Affiliations:** Department of Thermal and Fluids Engineering, University Carlos III of Madrid, 28911 Leganés, Spain; pagsalab@ing.uc3m.es; Tel.: +34-916249407

**Keywords:** catalyst layer, local resistance, ionomer, modeling, PEMFC

## Abstract

The optimized design of the catalyst layer (CL) plays a vital role in improving the performance of polymer electrolyte membrane fuel cells (PEMFCs). The need to improve transport and catalyst activity is especially important at low Pt loading, where local oxygen and ionic transport resistances decrease the performance due to an inevitable reduction in active catalyst sites. In this work, local oxygen and ionic transport are analyzed using direct numerical simulation on virtually reconstructed microstructures. Four morphologies are examined: (i) heterogeneous, (ii) uniform, (iii) uniform vertically-aligned, and (iv) meso-porous ionomer distributions. The results show that the local oxygen transport resistance can be significantly reduced, while maintaining good ionic conductivity, through the design of high porosity CLs (ε≃ 0.6–0.7) with low agglomerated ionomer morphologies. Ionomer coalescence into thick films can be effectively mitigated by increasing the uniformity of thin films and reducing the tortuosity of ionomer distribution (e.g., good ionomer interconnection in supports with a vertical arrangement). The local oxygen resistance can be further decreased by the use of blended ionomers with enhanced oxygen permeability and meso-porous ionomers with oxygen transport routes in both water and ionomer. In summary, achieving high performance at low Pt loading in next-generation CLs must be accomplished through a combination of high porosity, uniform and low tortuosity ionomer distribution, and oxygen transport through activated water.

## 1. Introduction

Polymer electrolyte membrane fuel cells (PEMFCs) are one of the most promising alternatives to internal combustion engines due to their high efficiency, high power density, low noise, and low pollutant emission (only water and heat are generated by electrochemical reactions) [1,2,3]. However, the high cost caused by the use of elevated Pt loadings at the cathode still remains as a critical issue for the widespread commercialization of PEMFCs either in portable, transport, or stationary applications [4,5]. As a result, a large body of current research is devoted to mitigating potential losses in low Pt loading electrodes (see, e.g., [6,7,8,9,10,11] and references therein).

Operation at low Pt loading is limited by the sharp increase in the so-called local oxygen transport resistance around Pt sites, which originates a high concentration overpotential at the cathode [12,13]. The origin of the resistance is inherently caused by the reduction in active Pt surface area at low Pt loading, so that the local flux toward each catalyst particle is amplified to maintain a targeted current density (per unit of geometric or platform area). In other words, the lower the active surface area, the higher the oxygen flux that each catalyst particle must support [14]. Therefore, electrochemical activity and transport in the neighbourhood of every catalyst particle must be optimized to mitigate the limitation (i.e., high flux demand) caused by a reduction in Pt loading [7]. The identification of suitable mitigation strategies is an essential step to meet the technological targets established for PEMFCs in the near term [6,15].

A large experimental effort has been accomplished in the last decades to reduce Pt loading around $0.1\;{\mathrm{mg}}_{\mathrm{Pt}}\;{\mathrm{cm}}^{-2}$. Some relevant experimental works dealing with the design of improved CLs at low Pt loading are reviewed below. In 2006–2012, Debe et al. [16,17] presented nanostructured thin film (NSTF) catalysts produced by 3M. Ultra-thin CLs ($\delta_{cl}≃0.3\;\mu m$) were prepared with a Pt monolayer supported on crystalline organic whiskers without an ionomer binder. Proton and electron conduction relayed in liquid water and Pt catalyst, respectively. High performance was reported for low Pt loading ($≃0.1\;{\mathrm{mg}}_{\mathrm{Pt}}\;{\mathrm{cm}}^{-2}$) with superior stability in a cyclic durability test compared to a conventional Pt/C catalyst. The better performance at low Pt loading was ascribed to the absence of ionomer and the agglomerated Pt/whisker structure, which reduced Pt growth and coalescence due to Ostwald ripening. Yu et al. (2015) [18] examined the feasibility of the reactive spray deposition technique for the preparation of high-performance CLs. They reported a better distribution of ionomer in active Pt sites and ionomer penetration into nanopores with a pore size of 1.7–10 nm. As a result, the optimal ionomer-to-carbon weight ratio was decreased from around 0.65 for a conventional CL down to 0.3. The improved ionomer distribution and lower ionomer content led to higher performance at low Pt loading. Talukdar et al. (2019) [19] studied the effect of drying on the preparation of conventional ink-based CLs. They found that solvent removal via sublimation by means of freeze drying led to a 3.5-fold increase in the effective porosity, thereby enhancing performance at low Pt loading ($0.16\;{\mathrm{mg}}_{\mathrm{Pt}}\;{\mathrm{cm}}^{-2}$). Conde et al. (2019) [20] analyzed the mass transport and water uptake properties of CLs prepared by electrospry. A significant decrease in the local oxygen transport resistance was reported compared to conventional CLs prepared by airbrushing ($0.8\;s\;{\mathrm{cm}}^{-1}$ vs. $0.2\;s\;{\mathrm{cm}}^{-1}$ at $0.025\;{\mathrm{mg}}_{\mathrm{Pt}}\;{\mathrm{cm}}^{-2}$, full humidification, and $80{\;}^{\circ }C$). Moreover, electrosprayed CLs showed a much higher water uptake than conventional CLs at the micro-/meso-scale. Yoshino et al. (2020) [21] presentedan ionomer nanofiber scaffolding CL formed by the combination of electrospinning of Nafion nanofibers and electrospraying of a catalyst ink on nanofibers. The multiscale ionomer microstructure showed improved performance at low RH and reduced catalyst poisoning thanks to the decrease in the ionomer content in the catalyst ink. More recently, Cheng et al. (2022) [22] produced CLs with nanoporous ionomer using polyvinyl alcohol as a sacrificial pore-forming agent during CL preparation. Water-filled pores in ionomer allowed a dramatic reduction in the local oxygen transport resistance from $0.37\;s\;{\mathrm{cm}}^{-1}$ down to $0.08\;s\;{\mathrm{cm}}^{-1}$. Zhang et al. (2022) [23] developed porous ionomers by incorporating ionic covalent organic framework nanosheets into Nafion. Meso-porosity significantly enhanced oxygen permeation and mass activity, increasing performance at low Pt loading by a factor of 1.6 compared to conventional CLs.

The small nanometric dimensions found in CLs make mumerical modeling especially important to understand and develop new microstructural designs [24]. An increasing body of numerical work has been presented in the literature dealing with the modeling of CLs by molecular-scale techniques, such as molecular dynamics (MD) by pore-scale techniques, direct numerical simulation (DNS), and pore network modeling (PNM), or by macroscopic techniques, such as volume averaging. Zenyuk et al. (2016) [25] analyzed the effect of CL thickness on performance using a 2D multiphase macroscopic model, with a focus on water transport in thin CLs. They observed that water removal via phase-change-induced-flow is significantly decreased in CLs below 5μm in thickness, leading to severe performance drop. Water flooding at the cathode can be mitigated by enhancing water removal though the anode and/or reducing the water retention capacity of the cathode CL. Mu et al. (2019) [26] modeled the effect of low Pt loading on local gas species transport using the lattice Boltzmnann method (LBM) on reconstructed CL microstructures. They concluded that the local species transport resistance is mainly caused by the high diffusion resistance of ionomer film, which is around 5.9% of the bulk transport resistance in a membrane. The local gas species transport resistance increases with I/C due to the formation of thicker ionomer films and decreases with porosity due to the growth of the ratio of ionomer loading relative to Pt. In a subsequent work, Mu et al. (2021) [27] investigated the effect of liquid water saturation and Pt distribution on local oxygen transport resistance considering a multiphase version of the LBM model presented in [26]. They showed that the effective oxygen diffusivity significantly decreases in hydrophilic CLs, simultaneously reducing the effective ionomer surface area and pore space available for transport. As a result, the local oxygen transport resistance increases in hydrophilic CLs, especially when oxygen permeation through liquid water is ignored. Lee et al. (2022) [28] examined water, proton, and oxygen transport in thin ionomer films of varying thicknesses by means of MD. They found that thick ionomer films are favorable to improve water and proton transport, while thin ionomer films enhance oxygen transport due to increased solubility in ionomer. Dou et al. (2022) [29] investigated the effect of capillary condensation on effective oxygen diffusivity and proton conductivity using LBM. They concluded that oxygen blockage by liquid water can be significantly suppressed with hydrophobic CLs. Moreover, the results showed the vital role of liquid water on proton conduction, reaching a better agreement with previous data when the contribution of water conduction was taken into account. Recently, Sadeghi et al. (2024) [30] used PNM to model transport in fresh and aged CLs, which accounted for four phases, namely void, ionomer, carbon, and catalyst. Good agreement was found with experimental data, considering the reduction in the electrochemical surface area (ECSA) originated by degradation. The results highlighted the importance of CL microstructure in PEMFC performance.

In the last few years, a growing interest has been devoted to understanding the impact of heterogeneous CL composition on both performance and durability. In this context, numerical modeling provides an efficient tool to asses experimental trends in CLs with different multi-component microstructures and heterogeneous morphologies. Mu et al. (2022) [31] used a multiscale version of the models presented in [26,27] to examine reactive transport of oxygen and water in four reconstructed agglomerates in the cathode CL. The results showed that the local oxygen transport resistance increases linearly with ionomer content and exponentially with uncatalyzed carbon volume fraction. Furthermore, the mean size of primary pores increases with porosity, a critical aspect to minimize the local oxygen transport resistance. García-Salaberri et al. (2022) [12] presented a 1D + 1D multiscale macroscopic model of an optimized cathode CL, in which ionomer pillars where covered by a meso-porous shell of electronically conductive material. They concluded that local oxygen transport resistance can be significantly suppressed by allowing transport of oxygen through activated water-filled meso-pores, without incurring significant ohmic losses. Dou et al. (2023) [32] analyzed the effect of carbon aggregation and ionomer morphology on performance by means of a LBM model. They found that a reasonable degree of agglomeration of carbon support can provide an optimal balance between pore and cross-ionomer transport resistances. In addition, a uniform ionomer coating is favourable to further improve performance, being the optimal ionomer content determined by a proper balance between the ECSA and the oxygen transport resistance.

The above experimental and numerical literature review shows the crucial role that multi-component composition and morphology play on transport in CL and therefore PEMFC performance. The aim of this work is to examine numerically the impact of the most relevant approaches identified in the literature to mitigate local transport resistances at low Pt loading, namely ionomer content, distribution, and porosity. Diffusion and conduction simulations are performed at the intra-agglomerate scale to evaluate both the local oxygen and the ionic transport resistances around catalyst sites. The predictions are systematically compared with previous observations to shed new light on key factors to be considered for improving performance at low Pt loading. The organization of this paper is as follows. In Section 2, the generation algorithm used for the virtual reconstruction of CL morphologies and the numerical model used for the calculation of local oxygen and ionic transport resistances are presented. The results are discussed in Section 3, which includes a calibration of the numerical model and a parametric analysis of volume composition, carbon/ionomer interaction, oxygen diffusivity in ionomer, and meso-porous ionomer. Finally, the conclusions are presented in Section 4.

## 2. Methods

### 2.1. Virtual Reconstruction

**Figure 1:**
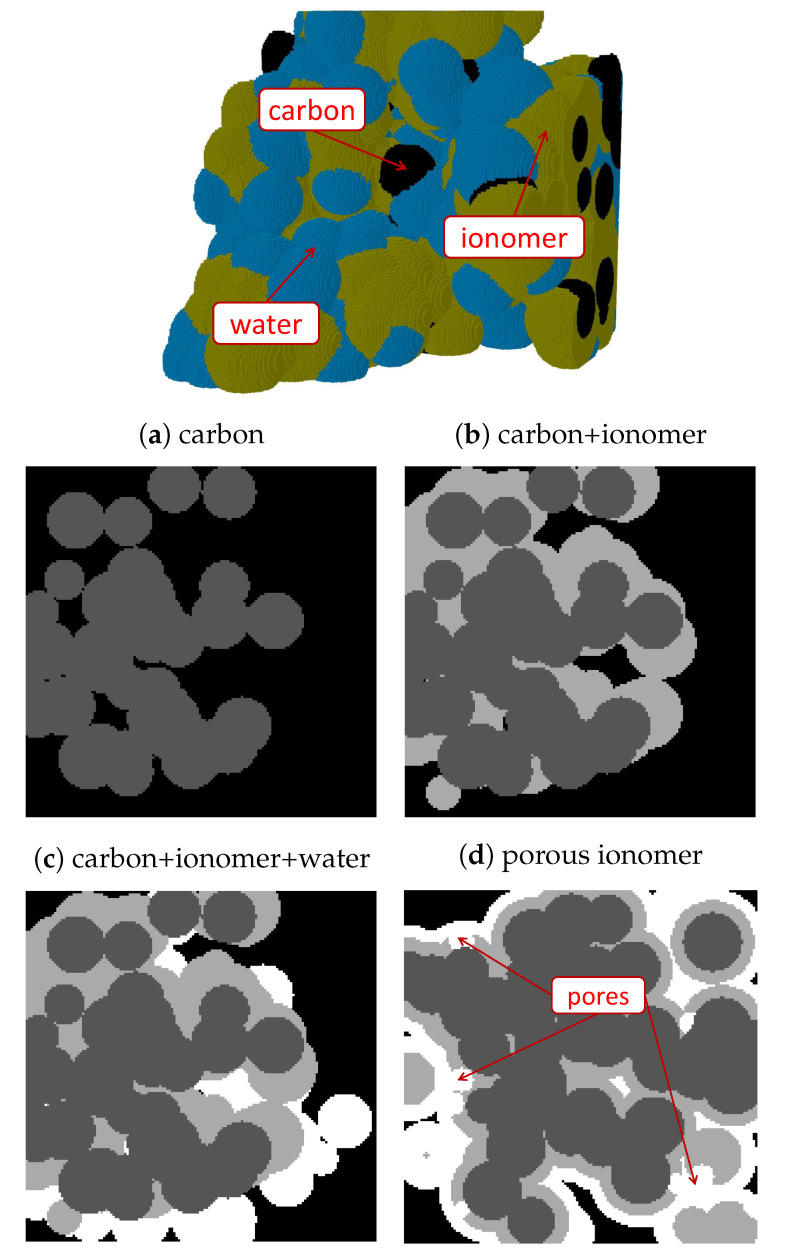
3D representation of carbon (black), ionomer (orange), and water (blue) distributions in a virtually reconstructed heterogeneous CL, and steps followed in the generation algorithm: (**a**) spherical carbon particles (black) are added randomly to form an agglomerated structure that joins the bottom and top surfaces of the image, (**b**) ionomer (light grey) is randomly added in semi-spherical regions around carbon particles, and (**c**) water (white) is added in semi-spherical regions around ionomer films. (**d**) Spherical pores are additionally incorporated into ionomer to create meso-porous ionomer.

CL microstructure was virtually reconstructed to differentiate among three phases, carbon, ionomer, and free water, considering four ionomer morphologies: (i) heterogeneous, (ii) uniform, (iii) idealized vertically-aligned, and (iv) meso-porous ionomer distributions. The size of the representative domain was set to $300\times 300\times 300\;{\mathrm{nm}}^{3}$, significantly lower than the average CL thickness, typically around $\delta_{cl}∼10\;\mu m$. The voxel resolution was kept equal to 2nm throughout the work. Figure 1 shows examples of the steps followed for the generation of heterogeneous CL microstructures, along with final modifications added to create meso-porous ionomer. Uniform and idealized morphologies were omitted in Figure 1 for brevity since they involve small changes in the algorithm used for the heterogeneous case. The main steps followed in the generation process were as follows:

1. **Carbon agglomeration.** As shown in Figure 1a, spherical carbon particles were randomly located in the domain with a uniform radius of $r_{c}=25\;\mathrm{nm}$, allowing for overlap between them. Particles were incorporated one by one into the domain until a prescribed carbon volume fraction, $\varepsilon_{c}$, was reached. After each particle addition, only the largest connected component was maintained in the process, while isolated particles not connected to the main carbon structure were removed. At the end, the connectivity of the agglomerated carbon structure to the top and bottom surfaces of the domain was checked, and the generation process was repeated from the beginning if there was not a connected pathway across the domain (six-connected voxels criterion). Usually, no more than five iterations were needed to reach a connected structure at the lowest carbon volume fraction examined, $\varepsilon_{c}=0.2$. The generation of the idealized carbon support composed of vertically-aligned cylinders was accomplished using a simplified algorithm. Carbon cylinders were placed with a uniform spacing in the material plane and the radius increased until reaching a prescribed carbon volume fraction.
2. **Ionomer addition.** As shown in Figure 1b, a heterogeneous ionomer was created by randomly selecting points from the carbon agglomerate and introducing semi-spherical films around the structure. Ionomer films were incorporated one at a time by identifying the void voxels enclosed in a sphere centered at the selected carbon point with a prescribed ionomer radius, $r_{i}$. For each radius, ionomer films were sequentially added until no further variation of the ionomer volume fraction, $\varepsilon_{i}$, was detected (below an established threshold). The whole process was completed when a prescribed porosity, ε, was reached, gradually increasing $r_{i}$ by a factor of 1.2 from an exceedingly small value ($r_{i}=2\;\mathrm{nm}$). For uniform coating, the ionomer phase was simply identified using the Ecludiean distance transform, so that void voxels located at a distance below $r_{i}$ from the carbon phase were identified as ionomer. As in the heterogeneous case, the ionomer radius was gradually increased by a factor of 1.2 from $r_{i}=2\;\mathrm{nm}$ until reaching the prescribed porosity, ε. In all cases, connectivity was checked after every ionomer addition to remove isolated components not connected to the main carbon+ionomer structure.
3. **Free water addition.** As shown in Figure 1c, free water was added in a similar way to ionomer. However, random points were selected from either carbon, ionomer, or water phases to identify void voxels to be converted into free water. The radius of water spheres, $r_{w}$, was sequentially increased by a factor of 1.2 from the last ionomer radius used in Step 2 until reaching a prescribed water saturation, *s*. In structures with uniform morphology, water was placed uniformly around ionomer by gradually increasing $r_{w}$ by a factor of 1.2. Isolated water blobs which were not connected to carbon or ionomer were removed.
(4) **Additional features.** Modifications of the morphology were incorporated to include specific features in the carbon, ionomer, and water distributions. As shown in Figure 1d, meso-porous ionomer was created by introducing water-filled spherical pores in the ionomer phase with a prescribed radius equal to the last ionomer radius used in Step 2. A total of 120 random points were selected from the ionomer phase.

### 2.2. Numerical Model

Oxygen diffusion and ionic conduction were examined by DNS of mass species and charge conservation equations, i.e., Laplace equation, at pore scale (see, e.g., [33,34])

(1) $$\nabla \cdot \left(\Gamma \nabla \varphi \right)=0$$

where $\Gamma =D_{O_{2}}^{\mathrm{eff}}\;\left[m^{2}\;s^{-1}\right],\sigma_{p}^{\mathrm{eff}}\;\left[S\;m^{-1}\right]$ is the property of interest, either mass diffusivity or ionic conductivity, and $\varphi =C_{O_{2}}\;\left[\mathrm{mol}\;m^{-3}\right],\phi_{p}\;\left[V\right]$ is the variable of interest, either oxygen concentration or ionic potential.

**Figure 2:**
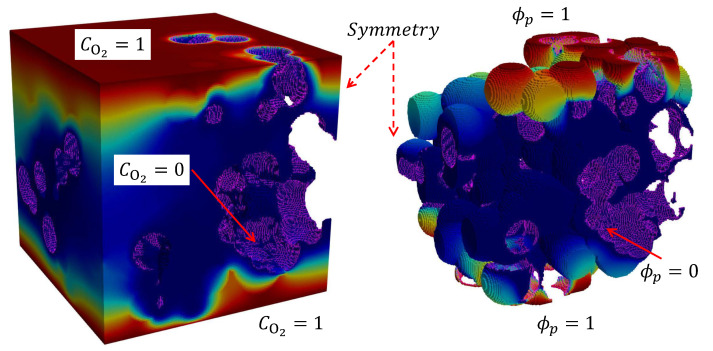
Schematic of the boundary conditions used in the calculation of $O_{2}$ and ionic transport resistances. Dirichlet boundary conditions are prescribed in the upper and lower exterior surfaces of the domain ($C_{O_{2}}=1,\;\phi_{p}=1$) and the interior ionomer/carbon surface (magenta) where the reaction takes place ($C_{O_{2}}=0,\;\phi_{p}=0$)—a water/carbon interface is also considered for meso-porous ionomer. Symmetry conditions are set at sidewalls of the representative domain. The void space was removed in the distribution corresponding to the ionic transport resistance since it is meaningless.

The generated microstructures were imported into the CFD software ANSYS Fluent 2020 R1 using a numerical mask to identify carbon, ionomer, free water, and void regions in a hexahedral mesh created with the same voxel resolution of image stacks. The various cell regions were then differentiated in the numerical mesh using the built-in capabilities of ANSYS fluent to separate cell regions and create the corresponding interfaces. Simulations were carried out on ionomer, water, and void phases, considering a no-flux boundary condition at passive carbon interfaces where reaction does not take place. As shown in Figure 2, a Dirichlet boundary condition was set at the reactive carbon/ionomer interface, corresponding to a limiting current density condition ($C_{O_{2}}=\phi_{p}=0$). Additionally, the carbon/water interface was also assumed to be reactive ($C_{O_{2}}=\phi_{p}=0$) in simulations with meso-porous ionomer. The system was excited by introducing a concentration or potential difference at the upper and lower surfaces ($C_{O_{2}}=\phi_{p}=1$). Symmetry boundary conditions were set at sidewalls to mimic the representative location of the domain within larger carbon agglomerated structures.

The diffusivity and the ionic conductivity of ionomer and water were set as constant. In the void phase, diffusivity was corrected for Knudsen diffusion based on the average pore radius (determined by applying the Euclidean distance transform on image stacks). Ionic conductivity of the void phase was set to a negligible value due to the absence of conductive routes for protons. According to previous work, the values adopted in each region were as follows

(2a) $$D_{O_{2}}^{\mathrm{eff}}=\left\{\begin{array}{cc} 3\times 10^{-11}\;m^{2}\;s^{-1} & \mathrm{ionomer}\\ 4\times 10^{-9}\;m^{2}\;s^{-1} & \mathrm{water}\\ \frac{D_{O_{2}}^{\mathrm{bulk}}}{1+\frac{D_{O_{2}}^{\mathrm{bulk}}}{D_{O_{2}}^{kn}}} & \mathrm{void} \end{array}\right.$$

(2b) $$\sigma_{p}^{\mathrm{eff}}=\left\{\begin{array}{cc} 1\;S\;m^{-1} & \mathrm{ionomer}\\ 0.1\;S\;m^{-1} & \mathrm{water}\\ 10^{-3}\;S\;m^{-1} & \mathrm{void}\;(\mathrm{assumed}) \end{array}\right.$$

Comparatively, the effective oxygen diffusivity in ionomer ($D_{O_{2},i}^{\mathrm{eff}}∼10^{-7}\;{\mathrm{cm}}^{2}\;s^{-1}$ [26,27,35,36]) is around five and two orders of magnitude lower than that in void space ($D_{O_{2},v}^{\mathrm{eff}}∼10^{-2}\;{\mathrm{cm}}^{2}\;s^{-1}$ [12,14]) and liquid water ($D_{O_{2},w}^{\mathrm{eff}}∼10^{-5}\;{\mathrm{cm}}^{2}\;s^{-1}$ [12,37]). Hence, void space and liquid water lead to a negligible concentration drop compared to ionomer, which is where almost the entire oxygen transport resistance originates [14,26]. In contrast, the effective ionic conductivity of ionomer ($\sigma_{p}^{\mathrm{eff}}∼1\;S\;m^{-1}$ [38,39,40,41]) is around one order of magnitude higher than that of liquid water ($\sigma_{p}^{\mathrm{eff}}∼0.1\;S\;m^{-1}$ [12,42,43]). The increase in the effective ionic conductivity of ionomer (one order of magnitude) is not as pronounced as the decrease in the effective oxygen diffusivity (two orders of magnitude).

In the above expressions, $D_{O_{2}}^{\mathrm{bulk}}$ and $D_{O_{2}}^{kn}$ are the bulk and Knudsen diffusivities of oxygen, given by [12,14]

(3a) $$\begin{array}{cccccc} & D_{O_{2}}^{\mathrm{bulk}} & & =2.65\times 10^{-5}{\left(\frac{T}{333.15}\right)}^{1.5} & & m^{2}\;s^{-1} \end{array}$$

(3b) $$\begin{array}{cccccc} & D_{O_{2}}^{kn} & & =\frac{r_{v}}{3}\sqrt{\frac{8RT}{\pi M_{O_{2}}}} & & m^{2}\;s^{-1} \end{array}$$

where $r_{v}$ is the average pore radius of void space, *T* is the temperature (fixed to $80{\;}^{\circ }C$), $M_{O_{2}}$ is the molecular mass of oxygen, and *R* is the universal gas constant.

The local mass and ionic transport resistances per unit of geometric (or platform area of the CL), $R_{O_{2}}^{\mathrm{local}}$ and $R_{p}^{\mathrm{local}}$, are obtained from a flux balance [14]

(4a) $$\begin{array}{cccc} & N_{O_{2},\mathrm{Pt}}^{\mathrm{local}}r_{f}=\frac{\Delta C_{O_{2}}}{R_{O_{2}}^{\mathrm{local}}} & & \Rightarrow R_{O_{2}}^{\mathrm{local}}=\frac{\Delta C_{O_{2}}}{N_{O_{2},\mathrm{Pt}}^{\mathrm{local}}}\frac{1}{r_{f}} \end{array}$$

(4b) $$\begin{array}{cccc} & N_{p,\mathrm{Pt}}^{\mathrm{local}}r_{f}=\frac{\Delta \phi_{p}}{R_{p}^{\mathrm{local}}} & & \Rightarrow R_{p}^{\mathrm{local}}=\frac{\Delta \phi_{p}}{N_{p,\mathrm{Pt}}^{\mathrm{local}}}\frac{1}{r_{f}} \end{array}$$

where $r_{f}=A_{\mathrm{Pt}}/A_{\mathrm{geo}}$ is the roughness factor, defined as the ratio between the ECSA and the CL geometric area, $N_{O_{2},\mathrm{Pt}}^{\mathrm{local}}$ and $N_{p,\mathrm{Pt}}^{\mathrm{local}}$ are the oxygen and proton flux at Pt surface, and $\Delta C_{O_{2}}$ and $\Delta \phi_{p}$ are the concentration and ionic potential drops from the bulk space toward Pt surface, respectively. $\Delta C_{O_{2}}$ was determined as the average concentration in the void space minus the oxygen concentration at reaction sites ($\Delta C_{O_{2}}=C_{O_{2}}^{\mathrm{bulk},\mathrm{avg}}$), while $\Delta \phi_{p}$ was directly taken as the prescribed potential drop ($\Delta \phi_{p}=1$). According to Equations (4a) and (4b), the local resistance per unit of the geometric area is inversely proportional to the roughness factor ($R^{\mathrm{local}}\propto r_{f}^{-1}$), dramatically increasing at low Pt loading when $A_{\mathrm{Pt}}\to 0$ [13,14,44].

Pt was assumed to be well dispersed over Pt surface, so no explicit description of the Pt nano-particle shape was made ($A_{c}≃A_{\mathrm{Pt}}$). Consequently, the roughness factor is meaningless, and the variable of interest calculated in the simulations was the local resistance per unit of Pt (or carbon) surface area $R_{\mathrm{Pt}}^{\mathrm{local}}$

(5a) $$\begin{array}{cccc} & R_{O_{2},\mathrm{Pt}}^{\mathrm{local}} & & =R_{O_{2}}^{\mathrm{local}}r_{f}=\frac{\Delta C_{O_{2}}}{N_{O_{2},\mathrm{Pt}}^{\mathrm{local}}} \end{array}$$

(5b) $$\begin{array}{cccc} & R_{p,\mathrm{Pt}}^{\mathrm{local}} & & =R_{p}^{\mathrm{local}}r_{f}=\frac{\Delta \phi_{p}}{N_{p,Pt}^{\mathrm{local}}} \end{array}$$

$R_{\mathrm{Pt}}^{\mathrm{local}}$ controls the incremental slope of $R^{\mathrm{local}}$ when the Pt loading (and $A_{\mathrm{Pt}}$) are decreased, removing the expected effect of the increase in $N_{\mathrm{Pt}}^{\mathrm{local}}$ with respect to $N^{\mathrm{local}}$ caused by area (i.e., $N_{\mathrm{Pt}}^{\mathrm{local}}=r_{f}N^{\mathrm{local}}$). The only concern is that the enlargement of transport pathways created by the discrete nature of Pt nano-particles at a given Pt loading is ignored [45]. Nevertheless, the aim of this work is to examine the overall effect of ionomer morphology, so the contribution of the discrete Pt shape is a secondary factor.

## 3. Discussion of Results

The discussion of results is divided into five sections. First, the model is calibrated in terms of local oxygen transport resistance and effective diffusivity of oxygen in ionomer. The results of a parametric analysis are then presented, which includes a study of volume composition, carbon/ionomer interaction, ionomer diffusivity, and meso-porous ionomer. The local oxygen and ionic transport resistances computed in the parametric analysis were averaged among five sample realizations for every condition examined. Water saturation was fixed to s=0.3, while porosity and carbon volume fraction were varied depending on the case under study.

### 3.1. Calibration

**Figure 3:**
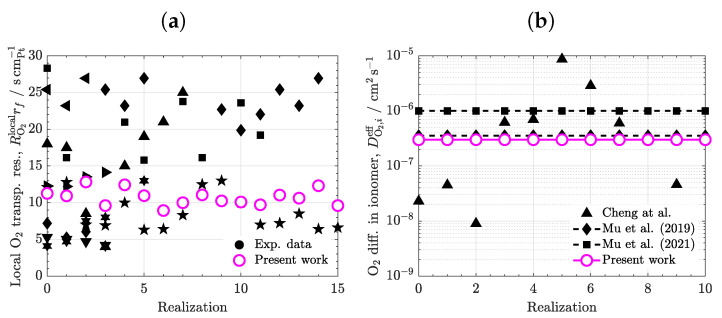
(**a**) Local $O_{2}$ transport resistance normalized with respect to Pt surface area, $R_{O_{2}}^{\mathrm{local}}r_{f}$, computed in simulations with a heterogeneous CL ($\varepsilon_{c}=0.3,\;\varepsilon =0.4$) compared to previous experimental data presented in the literature [49,50,51,52,53,54,55]. (**b**) Effective diffusivity coefficient of $O_{2}$ in ionomer, $D_{O_{2},i}^{\mathrm{eff}}$, used as a baseline value in the work compared to previous experimental data reported for Nafion [35] and numerical data considered in pore-scale simulations [26,27]. Black symbols show data from previous literature sources.

Before proceeding further, the predictive capabilities of the model were compared against data reported in the literature. The variables considered for the model calibration were the local oxygen transport resistance, $R_{O_{2}}r_{f}$, and the effective diffusivity of oxygen in ionomer, $D_{O_{2},i}^{\mathrm{eff}}$, due to the uncertainty in mass transport results and the availability of previous experimental data. Figure 3a shows the experimental data of $R_{O_{2}}r_{f}$ reported by different literature sources, along with the numerical results computed with the baseline $D_{O_{2},i}^{\mathrm{eff}}$ used in the simulation campaign ($D_{O_{2},i}^{\mathrm{eff}}=3\times 10^{-7}\;{\mathrm{cm}}^{-2}\;s^{-1}$). A partially saturated CL (s=0.3) with a conventional composition ($\varepsilon_{c}=0.3$, ε=0.4) was used in the simulations. A large dispersion is found among previous data, with $R_{O_{2}}r_{f}$ ranging between $5\;s\;{\mathrm{cm}}_{\mathrm{Pt}}^{-1}$ and $30\;s\;{\mathrm{cm}}_{\mathrm{Pt}}^{-1}$ (a factor of six). These variations can be mainly ascribed to microstructural differences that arise from volume composition and manufacturing techniques. The numerical results computed in sixteen sample realizations vary stochastically around $12\;s\;{\mathrm{cm}}_{\mathrm{Pt}}^{-1}$, which is close to the mean experimental value (≈$15\;s\;{\mathrm{cm}}_{\mathrm{Pt}}^{-1}$). Hence, the numerical results can be considered representative of the behavior of a conventional CL when the oxygen diffusivity is fixed at $D_{O_{2},i}^{\mathrm{eff}}\approx 3\times 10^{-7}\;{\mathrm{cm}}^{-2}\;s^{-1}$. As shown in Figure 3b, the assumed diffusivity is in line with previous data adopted in mesoscopic simulations by Mu et al. [26,27], who varied the oxygen diffusivity between $2.5\times 10^{-7}\;{\mathrm{cm}}^{-2}\;s^{-1}$ and $1\times 10^{-6}\;{\mathrm{cm}}^{-2}\;s^{-1}$. This range agrees with previous experimental data reported for the oxygen diffusivity in bulk Nafion membranes, even though there is a large variability among authors (three orders of magnitude) [35]. The large fluctuation of the diffusivity in bulk Nafion can be ascribed to conditioning, substrate interaction, and confinement of membranes [46,47,48]. The oxygen diffusivity considered here is a good approximation, which leads to realistic values of the local oxygen transport resistance.

### 3.2. Volume Composition

**Figure 4:**
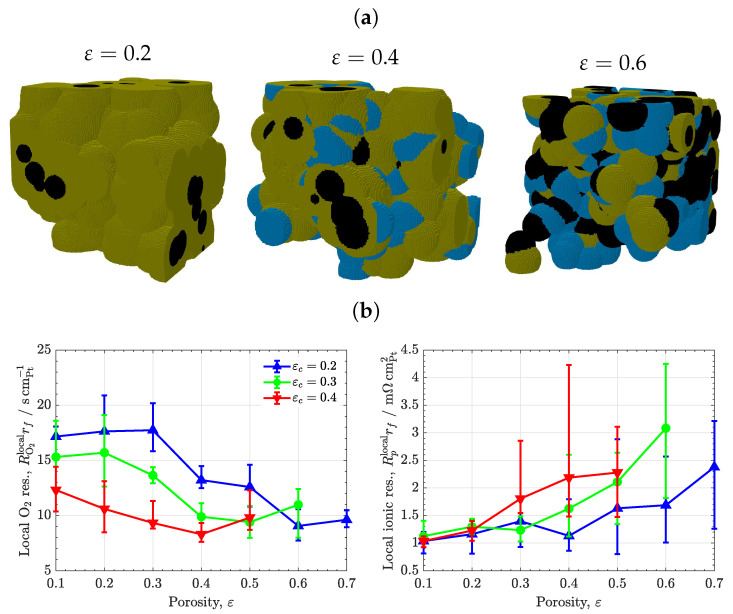
(**a**) Heterogeneous CL microstructures corresponding to $\varepsilon_{c}=0.3$ and various porosities, ε=0.2,0.4,0.6. Black: carbon, orange: ionomer, blue: water. (**b**) Variation of local $O_{2}$ and ionic transport resistances normalized with respect to Pt surface area, $R_{O_{2}}^{\mathrm{local}}r_{f}$ and $R_{p}^{\mathrm{local}}r_{f}$, as a function of porosity, ε, corresponding to three carbon volume fractions, $\varepsilon_{c}=0.2,0.3,0.4$. The error bars indicate the range of variation of results among different sample realizations.

Figure 4 shows the results of the analysis of the volume composition for conventional CLs with a heterogeneous ionomer distribution. The variation of the local oxygen and ionic transport resistances is examined as a function of porosity for three carbon volume fractions. As shown in Figure 4a, there is a strong dependency of $R_{O_{2}}r_{f}$ with porosity, which decreases by a factor of two when the porosity is increased from ε≃0.1 to ε≃0.7. That is, the ionomer volume fraction is reduced from $\varepsilon_{i}≃0.7$ to $\varepsilon_{i}≃0.1$, since $\varepsilon +\varepsilon_{i}+\varepsilon_{c}=1$. For a given porosity, $R_{O_{2}}r_{f}$ is also reduced with carbon volume fraction due to the decrease in ionomer volume fraction. These results agree with previous works, which showed that the use of moderate ionomer-to-carbon weight ratios (I/C≲0.65) is beneficial to enhance oxygen transport at low Pt loading due to a decrease in average ionomer thickness [14,18,44,53,56]. Notice also the non-linear dependency of the local oxygen transport resistance with porosity. For high ionomer volume fractions ($\varepsilon_{i}≃$ 0.5–0.7), $R_{O_{2}}r_{f}$ remains large and rather constant around $R_{O_{2}}r_{f}≳15\;s\;{\mathrm{cm}}_{\mathrm{Pt}}^{-1}$. In contrast, at lower ionomer volume fractions ($\varepsilon_{i}≃$ 0.1–0.5), $R_{O_{2}}r_{f}$ gradually decreases until it settles down around $R_{O_{2}}r_{f}≃10\;s\;{\mathrm{cm}}_{\mathrm{Pt}}^{-1}$ for CLs with low ionomer volume fractions and high porosities ($\varepsilon_{i}≃$ 0.1–0.3, ε≃ 0.5–0.7). Such non-linear behavior highlights the need to design high porosity CLs to mitigate the detrimental effect of local oxygen transport resistance at low Pt loading [57]. Experimentally, a strong reduction in $R_{O_{2}}r_{f}$ has been previously reported for high porosity electrosprayed CLs [20]. A low ionomer content minimizes the agglomeration of ionomer films, avoiding the formation of locally dense ionomer regions that prevent a proper distribution of oxygen throughout the ionomer surface. CL design with low ionomer fraction and high porosity must be accompanied by a moderate increase in the local ionic transport resistance. As shown in Figure 4b, $R_{p}^{\mathrm{local}}r_{f}$ remains rather constant around 1.5–2$\;m\Omega \;{\mathrm{cm}}_{\mathrm{Pt}}^{-2}$ for ε≲0.5 ($\varepsilon_{i}≳0.2$) but significantly increases beyond $3\;m\Omega \;{\mathrm{cm}}_{\mathrm{Pt}}^{-2}$ for ε≃0.7 ($\varepsilon_{i}≃0.1$) when ionomer interconnection approaches the percolation threshold [58]. Operation close to the ionic percolation threshold of ionomer must be ensured by the incorporation of supporting routes for proton transport, created, for example, by an enhanced water uptake at the micro- and meso-scale, as is the case for optimized electrosprayed CLs [20]. Alternatively, a delicate arrangement of ionomer might be necessary, as discussed in the next section.

### 3.3. Carbon/Ionomer Interaction

**Figure 5:**
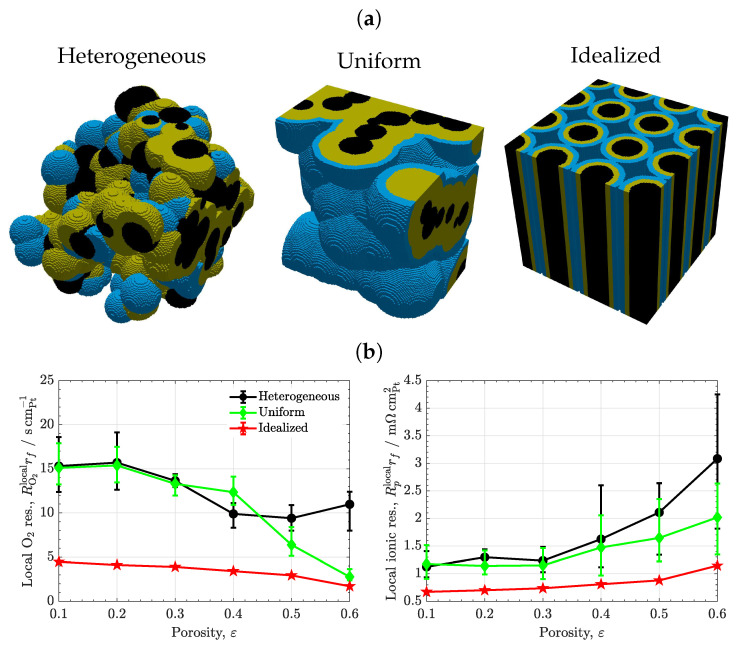
(**a**) Microstructures of CLs with heterogeneous, uniform, and idealized vertically-aligned ionomer morphologies ($\varepsilon_{c}=0.3,\;\varepsilon =0.5$). Black: carbon, orange: ionomer, blue: water. (**b**) Variations in local $O_{2}$ and ionic transport resistances normalized with respect to Pt surface area, $R_{O_{2}}^{\mathrm{local}}r_{f}$ and $R_{p}^{\mathrm{local}}r_{f}$, as a function of porosity, ε, corresponding to CLs to heterogeneous, uniform, and idealized vertically-aligned ionomer morphologies ($\varepsilon_{c}=0.3$). The error bars indicate the range of variation in results among different sample realizations.

**Figure 6:**
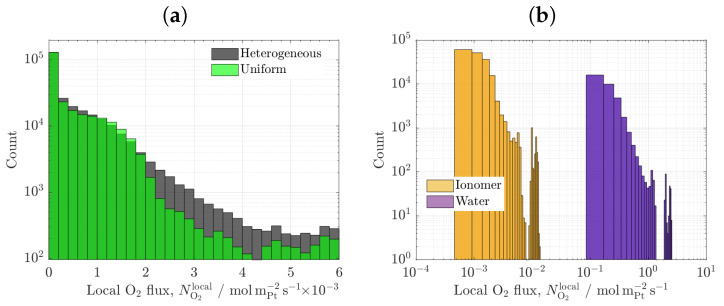
(**a**) Histograms of local $O_{2}$ flux at ionomer/carbon interface corresponding to CL microstructures with heterogeneous and uniform ionomer morphologies. (**b**) Histograms of local $O_{2}$ flux at ionomer/carbon and water/carbon interfaces corresponding to a CL with meso-porous ionomer. $\varepsilon_{c}=0.3,\;\varepsilon =0.4$.

Figure 5 shows the results of the carbon/ionomer interaction for three ionomer morphologies, heterogeneous, uniform, and idealized vertically aligned distributions, as a function of porosity. A representative carbon volume fraction is considered, $\varepsilon_{c}=0.3$. As shown in Figure 5a, the local oxygen transport resistance of the heterogeneous and uniform ionomer distributions are similar at low-to-middle porosities (ε≃ 0.1–0.4). The low sensitivity of $R_{O_{2}}r_{f}$ to ionomer heterogeneity is explained by the presence of both thick and thin ionomer regions in the heterogeneous samples, which lead on average to a similar oxygen resistance to that of uniform samples [59,60,61]. This situation is further illustrated in Figure 6a, which shows the histograms of the local oxygen flux at active sites of the examined samples at ε=0.4. For heterogeneous ionomer, the local oxygen flux distribution varies roughly linearly due to the presence of a continuous distribution of regions with high and low oxygen resistances. In contrast, for uniform ionomer, the local oxygen flux distribution is more concentrated toward intermediate values that are characteristic of the mean ionomer thickness (lower dispersion). The difference between heterogeneous and uniform ionomer distributions is enlarged at high porosity (ε≃ 0.5–0.6), where the uniform samples reach a lower oxygen resistance than the heterogeneous samples (a factor of two, $R_{O_{2}}r_{f}≃5\;s\;{\mathrm{cm}}_{\mathrm{Pt}}^{-1}$ vs. $R_{O_{2}}r_{f}≃10\;s\;{\mathrm{cm}}_{\mathrm{Pt}}^{-1}$). The reduction in the oxygen resistance at high porosity in the uniform samples is caused by a strong suppression of ionomer agglomeration when the ionomer volume fraction is low ($\varepsilon_{i}≃$ 0.1–0.2) [53]. The optimal arrangement of uniform ionomer virtually removes coalescence of ionomer films, facilitating oxygen distribution between ionomer films. Similarly, the local ionic transport resistance of the uniform samples is especially reduced compared to that of the heterogeneous samples at high porosity (a factor of two, $R_{p}r_{f}≃1.5\;m\Omega \;{\mathrm{cm}}_{\mathrm{Pt}}^{-2}$ vs. $R_{p}r_{f}≃3\;m\Omega \;{\mathrm{cm}}_{\mathrm{Pt}}^{-2}$). The superior ionic conductivity of uniform samples arises from the better arrangement of ionomer and the suppression of ineffective agglomerated ionomer regions, i.e., a tortuosity decrease [62]. Overall, the use of high porosity CLs with a uniform-as-possible ionomer distribution can be an effective way to improve performance at low Pt loading due to facilitated oxygen transport and good proton conduction [56,63,64].

The importance of ionomer morphology is exacerbated in the samples with an idealized vertically-aligned distribution. As shown in Figure 5a, the local oxygen transport resistance of the idealized samples is low in the full porosity range, slightly decreasing from $5\;s\;{\mathrm{cm}}_{\mathrm{Pt}}^{-1}$ to $2\;s\;{\mathrm{cm}}_{\mathrm{Pt}}^{-1}$ when the porosity is increased between 0.1 and 0.6. Furthermore, as shown in Figure 5b, the local ionic transport resistance is significantly reduced compared to the heterogeneous and uniform distributions, remaining between $0.6\;m\Omega \;{\mathrm{cm}}_{\mathrm{Pt}}^{-2}$–$1.1\;m\Omega \;{\mathrm{cm}}_{\mathrm{Pt}}^{-2}$ for ε = 0.1–0.6. The extremely high performance of the idealized ionomer distribution arises from a minimization in ionomer agglomeration and an optimal arrangement of proton transport pathways (i.e., tortuosity minimization) [12]. Notice that the oxygen resistance of the idealized and the uniform distributions are similar at high porosity, reflecting the superior oxygen distribution in both cases. High performance at low Pt loading with vertically aligned microstructures have been previously demonstrated in several experimental works [65,66,67], where current densities as high as $2.6\;A\;{\mathrm{cm}}^{-2}$ were reached at 0.6V with $0.1\;{\mathrm{mg}}_{\mathrm{Pt}}\;{\mathrm{cm}}^{-2}$ and air supply. As a final remark, it is worth noting that the results of the idealized morphology do not vary among sample realizations, unlike the results calculated with random carbon particles. This fact highlights the importance of using reproducible manufacturing techniques and homogenized catalyst supports to reduce maldistribution of transport resistances, which can affect local performance and degradation rates [68,69].

### 3.4. Ionomer Diffusivity

**Figure 7:**
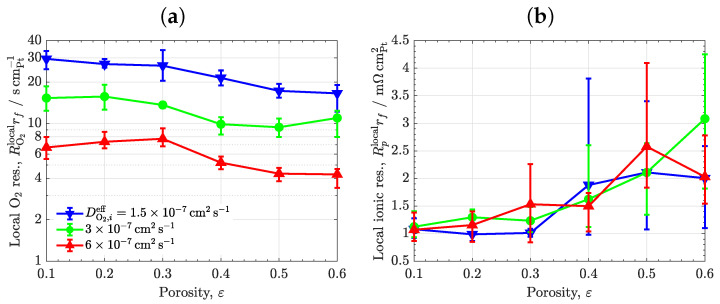
Variation of (**a**) local $O_{2}$, $R_{O_{2}}^{\mathrm{local}}r_{f}$, and (**b**) ionic. $R_{p}^{\mathrm{local}}r_{f}$, transport resistances as a function of porosity, ε, corresponding to three effective diffusivities of $O_{2}$ in ionomer, $D_{O_{2},i}^{\mathrm{eff}}=1.5\times 10^{-7}$, $3\times 10^{-7}$, $6\times 10^{-7}\;{\mathrm{cm}}^{2}\;s^{-1}$ ($\varepsilon_{c}=0.3$). The error bars indicate the range of variation of results among different sample realizations.

The results of the analysis of the effective oxygen diffusivity in ionomer are shown in Figure 7. The local oxygen and ionic transport resistances are plotted as a function of porosity for the heterogeneous ionomer distribution ($\varepsilon_{c}=0.3$). As expected, the local oxygen transport resistance decreases almost linearly with an increase in the oxygen diffusivity (see Figure 7a). Indeed, small deviations from linearity are caused by stochastic fluctuations that arise from random microstructures. As shown in Figure 7b, the local ionic transport resistance remains invariant with respect to oxygen diffusivity, reaching similar values to those presented in Figure 4b (except for stochastic variations among samples). The use of modified ionomers with enhanced permeability and good conductivity offers a viable route to improve performance at low Pt loading. Among available options, blended ionomers can be a satisfactory solution to produce tailored designs with balanced mass and ionic transport properties [70,71]. Nevertheless, it is worth noting that achieving a remarkable high performance may only be possible if enhanced diffusion is accompanied by a proper CL design in terms of high porosity and ionomer morphology. The increase in oxygen diffusivity shown in previous work is typical of order unity, so a disruptive increase in oxygen diffusivity may not be possible by solely altering the chemical ionomer structure [54,70,71,72,73]. Local oxygen transport can be more effectively increased by incorporating meso-porosity into ionomer, an aspect that is discussed in the next section.

### 3.5. Meso-Porous Ionomer

**Figure 8:**
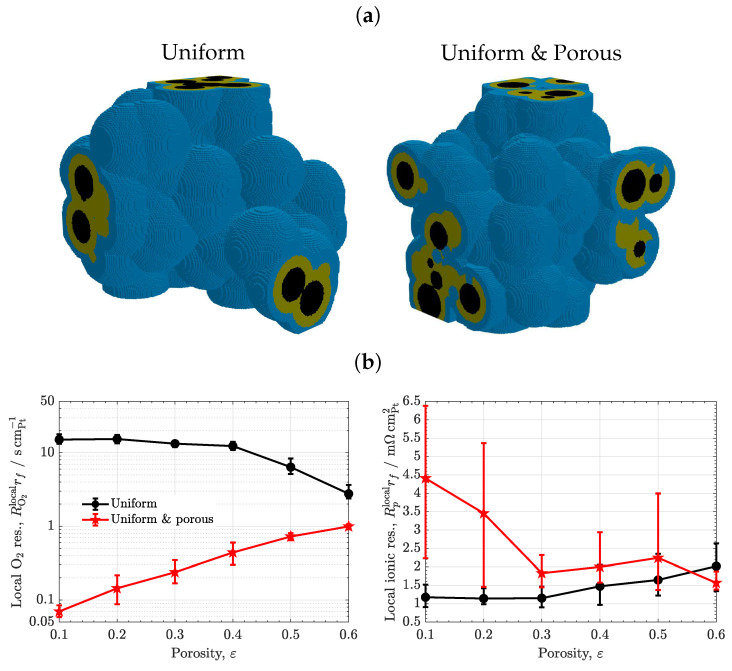
(**a**) Microstructures of CLs with uniform non-porous and uniform meso-porous ionomer morphologies ($\varepsilon_{c}=0.3,\;\varepsilon =0.5$). Black: carbon, orange: ionomer, blue: water. (**b**) Variation of local $O_{2}$ and ionic transport resistances normalized with respect to Pt surface area, $R_{O_{2}}^{\mathrm{local}}r_{f}$ and $R_{p}^{\mathrm{local}}r_{f}$, as a function of porosity, ε, corresponding to CLs with uniform non-porous and uniform meso-porous ionomer morphologies ($\varepsilon_{c}=0.3$). The error bars indicate the range of variation of results among different sample realizations.

The results of the uniform meso-porous ionomer are compared to those of the uniform ionomer as a function of porosity in Figure 8 ($\varepsilon_{c}=0.3$). As shown in Figure 8a, the local oxygen transport resistance of the meso-porous ionomer is sharply decreased, reaching values even one order of magnitude lower than the uniform ionomer ($R_{O_{2}}r_{f}≃0.1$–$1\;s\;{\mathrm{cm}}_{\mathrm{Pt}}^{-1}$ vs. $R_{O_{2}}r_{f}≃5$–$10\;s\;{\mathrm{cm}}_{\mathrm{Pt}}^{-1}$). The strong enhancement of oxygen transport is caused by the much higher diffusivity of oxygen in liquid water ($D_{O_{2},w}^{\mathrm{eff}}∼10^{-5}\;{\mathrm{cm}}^{2}\;s^{-1}$) compared to ionomer ($D_{O_{2},i}^{\mathrm{eff}}∼10^{-7}\;{\mathrm{cm}}^{2}\;s^{-1}$), two orders of magnitude higher—see histograms in Figure 6b. This result clearly evidences that oxygen resistance can be greatly suppressed by enhancing water uptake within ionomer and allowing oxygen transport directly through liquid water (provided that water regions are electrochemically activated). For instance, the increase in the oxygen resistance of the meso-porous ionomer with CL porosity is explained by a reduction in the carbon/water interface, since the ionomer pore radius was varied according to the film thickness (a decreasing function of CL porosity). As shown in Figure 8b, the local ionic transport resistance shows an opposite behaviour to that of the oxygen resistance, being higher in the meso-porous ionomer due to the worse transport of protons in liquid water (a factor around two to three, $R_{p}^{\mathrm{local}}r_{f}\approx 1.5$–$4.5\;m\Omega \;{\mathrm{cm}}_{\mathrm{Pt}}^{-2}$ vs. $R_{p}^{\mathrm{local}}r_{f}\approx 1$–$2\;m\Omega \;{\mathrm{cm}}_{\mathrm{Pt}}^{-2}$). The ionic resistance of the meso-porous ionomer decreases with porosity due to the presence of smaller water regions at high porosity (i.e., narrower ionomer film thickness). Comparatively, the increase in the ionic transport resistance of the meso-porous ionomer is significantly less important for performance than the reduction in the oxygen transport resistance, so reaching high performance with meso-porous ionomer is certainly possible. Previous experimental works that have shown improved performance at low Pt loading by exploiting the use of liquid water include (i) NSTF electrodes [16,17] and (ii) engineered porous ionomers [22,23,74,75]. In the latter group, it may also be included some blended ionomers with a significant bulky structure [70]. In agreement with the present results, a reduction in the local oxygen transport resistance down to one order of magnitude ($R_{O_{2}}=0.08\;s\;{\mathrm{cm}}^{-1}$ vs. $R_{O_{2}}=0.37\;s\;{\mathrm{cm}}^{-1}$) with a moderate ionic resistance in the polarization curve was recently reported by Cheng et al. [22] using a masked nanoporous ionomer. In addition, the low oxygen transport resistance of electrosprayed CLs at low Pt loading ($R_{O_{2}}^{\mathrm{local}}≃0.17\;s\;{\mathrm{cm}}^{-1}$) can be explained by the combination of high porosity, more uniform ionomer distribution, and enhanced water uptake at the micro-/meso-scale [20].

## 4. Conclusions

The local and ionic transport resistances of catalyst layers (CLs) used in polymer electrolyte membrane fuel cells (PEMFCs) have been analyzed by direct numerical simulation on virtually reconstructed microstructures. The generated samples accounted for carbon, ionomer, and liquid water, neglecting the discrete geometry of Pt nano-particles, to study the effect of volume composition, carbon/ionomer interaction, ionomer diffusivity, and ionomer meso-porosity. To this end, four ionomer morphologies were considered: (i) heterogeneous, (ii) uniform, (iii) idealized vertically-aligned, and (iv) meso-porous distributions. As summarized in Table 1, the numerical results have shown that the local oxygen transport resistance can be reduced through the design of high porosity CLs with low ionomer agglomeration. Increasing the uniformity and reducing the tortuosity of ionomer distribution avoids excessive coalescence of ionomer films, while enhancing proton transport. Oxygen transport can be further improved through the use of blended ionomers with high oxygen permeability and good ionic conductivity, as well as meso-porous ionomers. Allowing oxygen diffusion through liquid water near active Pt sites is crucial to strongly enhance oxygen transport toward Pt surface. Indeed, the high diffusivity of oxygen in liquid water compared to ionomer can decrease the oxygen resistance by one order of magnitude. The above guidelines, high porosity, uniform and low tortuosity ionomer, and oxygen transport through activated liquid water are key aspects to mitigate losses at low Pt loading in next-generation PEMFCs.

Several aspects warrant future work. The effect of the discrete geometry of Pt nano-particles and porous carbon support must be examined. In addition, a numerical analysis accounting for electrochemical reactions must be performed, with special emphasis on reaction kinetics in liquid water.

## Figures and Tables

**Table 1:** Comparison of the local oxygen and ionic transport resistances of the various ionomer morphologies examined in this work for a representative carbon volume fraction ($\varepsilon_{c}=0.3$). The relative variation with respect to the first case is shown in brackets.

Ionomer Morphology	Local O_2_ Resistance $\left[s\;{\mathrm{cm}}_{\mathrm{Pt}}^{-1}\right]$	Local Ionic Resistance $\left[m\Omega \;{\mathrm{cm}}_{\mathrm{Pt}}^{2}\right]$
Heterogeneous (ε= 0.1–0.3)	14.88 (–)	1.21 (–)
Heterogeneous (ε= 0.4–0.6)	10.09 (−32.19%)	2.27 (+87.6%)
Uniform (ε= 0.1–0.4)	14.03 (−5.71%)	1.23 (+1.65%)
Uniform (ε= 0.5–0.6)	4.57 (−69.29%)	1.83 (+51.24%)
Ideal (ε= 0.1–0.6)	3.42 (−77.02%)	0.82 (−31.92%)
Diffusivity increase (ε= 0.1–0.6)	5.93 (−60.15%)	1.64 (+35.9%)
Meso-porous (ε= 0.1–0.6)	0.43 (−97.08%)	2.58 (+113.22%)

## Data Availability

Data available on authors’ request.

## Nomenclature

***Symbols***	
*A*	area/m^2^
*C*	species concentration/mol m^−3^
*D*	mass diffusivity/m^2^ s^−1^
*M*	molecular mass/kg mol^−1^
*N*	flux/mol m^−2^ s^−1^ or A m^−1^
*R*	universal gas constant/J mol^−1^ K^−1^
*R_i_*	mass transport or ionic resistance/s m^−1^ or Ωm^2^
*r*	radius/m
*r_f_*	roughness factor/−
*s*	water saturation/−
*T*	temperature/K
***Greek letters***	
Г	diffusivity or conductivity/IS units
*δ*	thickness/m
*ε*	porosity/−
*ε_i_*	volume fraction of component *i*/−
*σ_p_*	ionic conductivity/S m^−1^
*ϕ_p_*	ionic potential/V
*φ*	transport scalar/IS units
***Subscripts***	
*c*	carbon
*cl*	catalyst layer
*i*	ionomer
*p*	protonic or ionic
*v*	void
***Superscripts***	
bulk	bulk property
eff	effective
*kn*	Knudsen
local	local quantity around active Pt sites
*w*	water
